# Cannabis use in Tunisian high school adolescents: MedSPAD 2021

**DOI:** 10.1192/j.eurpsy.2023.1122

**Published:** 2023-07-19

**Authors:** R. Mallekh, S. Rejaibi, A. Silini, M. Zid, I. Ben Slema, N. Zoghlami, S. Ben Youssef, M. Zribi, N. Ben Salah, H. Aounallah-Skhiri

**Affiliations:** 1 Department of Epidemiology, National Institute of Health; 2 Medical Faculty of Medicine, Tunis El Manar University; 3 Nutrition Surveillance and Epidemiology department, SURVEN Research Laboratory; 4 Intensive Care Unit department, Center for Urgent Medical Assistance, Tunis, Tunisia

## Abstract

**Introduction:**

Cannabis use is wrongly deemed “safe” by teenagers worldwide, despite its increased tetrahydrocannabinoids content and its psychosocial and cognitive harmful effectts.

**Objectives:**

We aimed to estimate the prevalence of cannabis use, identify associated factors among Tunisian adolescents and assess the risk of problem cannabis use among users.

**Methods:**

The 2021-Mediterranean School Survey Project on Alcohol and Other Drugs (MedSPAD) is a national survey, targeting Tunisian high school adolescents aged 16 to 18 years.

Based on a self-administered questionnaire, adolescents were asked about their engagement in several risky behaviours including cannabis use. The 6-item Cannabis Abuse Screening Test (CAST) was intended for users to assess the risk of problem cannabis use.

Binary logistic regression was performed to identify factors associated to cannabis use and Adjusted Odds Ratios (AORs) were presented with correspondent 95% confidence intervals (CI). Cspro and STATA software were used for data entry and analysis respectively.

**Results:**

Among 6201 participants (girls: 60.4%), lifetime prevalence of cannabis use was 7.9% ,95% CI [7.0, 8.9] significantly higher among boys (16.1 % Vs. 2.5 % in girls, p<10^-4^). Early onset (at 13 or younger) was reported by 8.6% of users.

Multivariate analysis showed that cannabis use was more prevalent in Tunis district(p=0.04), and significantly associated to alcohol, tobacco and electronic-cigarettes use (AOR of 6.2, 4.2 and 2.6 respectively, p<10^-3^). Absenteeism for non-medical reasons and nights spent away from home were also independent factors significantly associated with cannabis use (p<10^-3^).

The CAST indicated a high risk of cannabis-use-related problems in 67.2% of respondents (n=223).

**Conclusions:**

Cannabis use is increasingly common in Tunisian adolescents. Moreover, the alarming risk of problem cannabis use warrant the urgent need for school-based interventions and screening programs to prevent and control cannabis use especially among vulnerable subgroups.

**Disclosure of Interest:**

None Declared

